# Molecular Mechanisms Underlying NMDARs Dysfunction and Their Role in ADHD Pathogenesis

**DOI:** 10.3390/ijms241612983

**Published:** 2023-08-19

**Authors:** Justyna Kuś, Kamil Saramowicz, Maria Czerniawska, Wojciech Wiese, Natalia Siwecka, Wioletta Rozpędek-Kamińska, Aleksandra Kucharska-Lusina, Dominik Strzelecki, Ireneusz Majsterek

**Affiliations:** 1 Department of Clinical Chemistry and Biochemistry, Medical University of Lodz, Mazowiecka 5, 92-215 Lodz, Poland; justyna.kus1@stud.umed.lodz.pl (J.K.); kamil.saramowicz@stud.umed.lodz.pl (K.S.); maria.czerniawska@stud.umed.lodz.pl (M.C.); wojciech.wiese@stud.umed.lodz.pl (W.W.); natalia.siwecka@stud.umed.lodz.pl (N.S.); wioletta.rozpedek@umed.lodz.pl (W.R.-K.); ola_kucharska@wp.pl (A.K.-L.); 2 Department of Affective and Psychotic Disorders, Medical University of Lodz, Czechoslowacka 8/10, 92-216 Lodz, Poland; dominik.strzelecki@umed.lodz.pl

**Keywords:** N-methyl-D-aspartate receptor (NMDAR), attention deficit hyperactivity disorder (ADHD), synaptic plasticity, neurodevelopment, glutamate, long-term potentiation, long-term depression, genetic variants

## Abstract

Attention deficit hyperactivity disorder (ADHD) is one of the most common neurodevelopmental disorders, although the aetiology of ADHD is not yet understood. One proposed theory for developing ADHD is N-methyl-D-aspartate receptors (NMDARs) dysfunction. NMDARs are involved in regulating synaptic plasticity and memory function in the brain. Abnormal expression or polymorphism of some genes associated with ADHD results in NMDAR dysfunction. Correspondingly, NMDAR malfunction in animal models results in ADHD-like symptoms, such as impulsivity and hyperactivity. Currently, there are no drugs for ADHD that specifically target NMDARs. However, NMDAR-stabilizing drugs have shown promise in improving ADHD symptoms with fewer side effects than the currently most widely used psychostimulant in ADHD treatment, methylphenidate. In this review, we outline the molecular and genetic basis of NMDAR malfunction and how it affects the course of ADHD. We also present new therapeutic options related to treating ADHD by targeting NMDAR.

## 1. Introduction

Attention deficit hyperactivity disorder (ADHD) is one of the most frequent disorders in child and adolescent psychiatry [1]. According to the International Classification of Diseases 11th Revision (ICD-11), ADHD is characterized by recurrent patterns of impulsivity, hyperactivity, and inattention that persist for a minimum of six months and significantly impair daily functioning. Symptoms of ADHD typically appear before the age of 12 but can be diagnosed later in adolescence or adulthood when demands exceed coping capacities. Inattention refers to significant difficulty in concentration on tasks that do not provide a high level of stimulation or frequent rewards [2]. Individuals with ADHD often struggle to focus on tasks or instructions, frequently becoming easily distracted, which hinders their academic performance [3]. Hyperactivity, on the other hand, refers to excessive motor activity and difficulties with maintaining quiet engagement in activities, most evident in structured situations that require behavioural self-control. Impulsivity is defined as a tendency to act in response to immediate stimuli, without deliberation or consideration of the risks and consequences. The preponderance of specific symptoms allows for the classification of ADHD into three primary presentations: predominantly inattentive, predominantly hyperactive-impulsive, and combined type [2]. ADHD is associated with a higher risk of many mental disorders, such as depression or alcohol use disorders [4,5]. Moreover, emotional dysregulation in ADHD patients affects social interactions and close relationships [6]. Despite its relatively high prevalence of over 5%, the actual aetiology of ADHD remains unknown, probably due to its multifactorial background. Multiple genes, prenatal, and perinatal factors have been indicated as risk factors for developing ADHD [7]. Numerous studies have found a strong genetic association with structural and functional changes in the brain, as well as delayed neurodevelopment in ADHD (reviewed in [8]).

Executive deficits in ADHD are associated with widespread changes in several brain regions, such as the prefrontal cortex (PFC), temporal region, basal ganglia, and hippocampus [9,10,11]. Changes in the distribution of neurotransmitters in ADHD include abnormalities in dopamine, norepinephrine, serotonin, gamma-aminobutyric acid (GABA), and glutamate levels [12,13,14]. While dopamine deficit is the best-known factor associated with ADHD, an increasing amount of evidence indicates that disrupted glutamatergic transmission also plays a significant role in the disorder pathogenesis [15,16]. Studies have shown that people with ADHD have higher levels of glutamate in the striatum [17]. In the anterior cingulate cortex (ACC), the glutamate signal is increased in paediatric patients and decreased in adults with ADHD; this suggests neurodevelopmental changes in frontostriatal glutamatergic circuits over the lifespan of ADHD patients [18]. Glutamatergic dysfunction in ACC positively correlates with core ADHD symptoms, impulsivity, and hyperactivity [19]. Glutamate signalling is also a significant regulator in ADHD treatment, as it is necessary for behavioural sensitisation to methylphenidate (currently the most widely used psychostimulant in ADHD treatment) [20]. There is also evidence that several medications directly involved in glutamatergic signalling might be useful in the treatment of compulsivity and impulsivity in paediatric patients [21]. Glutamate is responsible for neuronal excitability by activating both ionotropic and metabotropic receptors. The N-methyl-D-aspartate receptor (NMDAR) is a ligand-gated ion channel that is predominantly involved in regulating synaptic plasticity, memory, and other cognitive functions [22]. More specifically, NMDARs abundantly expressed in CA1 and CA3 regions of the hippocampus are required for the acquisition of both spatial and working memory [23,24]. In the PFC, NMDARs are crucial for attention control and their hypofunction can lead to cognitive decline and attention deficits [25]. Furthermore, proper NMDAR activity finely tunes noradrenergic signalling in the locus coeruleus, which is essential for regulating attention, impulsivity, and exploratory behaviours [26]. Also, NMDAR deletion in dopaminergic neurons causes reduced phasic dopamine release contributing to learning deficits [27]. Altogether, the mentioned studies highlight the significant impact of proper NMDAR functioning on behavioural flexibility.

The International Multi-centre ADHD Gene (IMAGE) project provided comprehensive analysis of over 50 candidate genes that substantially contribute to ADHD susceptibility. The majority of these genes are involved in the regulation of dopaminergic, noradrenergic, and serotonergic pathways [28]. However, given the growing recognition of the genes involved in regulation of glutamatergic signalling and synaptic plasticity [29,30], the potential causal role of NMDAR dysfunction in the pathogenesis of ADHD has become a new area of research interest. For this reason, this review paper is aimed to focus on the role of NMDAR dysregulation in ADHD pathophysiology, which to date has not been studied extensively and is not well-understood. NMDAR dysfunction is one of the suggested mechanisms underlying ADHD pathogenesis in humans [31], as the role of NMDAR dysregulation has been well-established in a rat model of ADHD [32,33]. The most direct evidence is possibly from patients with a rare autoimmune disorder, anti-NMDAR encephalitis, in which autoantibodies against NMDAR cause NMDAR hypofunction. Some symptoms of affected children include inattention, hyperactivity, and impulsivity, which are hallmark characteristics of ADHD [34]. Research regarding NMDAR role in ADHD might be useful in the development of novel treatment strategies, as currently most effective drugs, atomoxetine and methylphenidate, beside inhibiting monoamine reuptake, also influence NMDAR function. Low doses of methylphenidate selectively enhance NMDAR response, whereas high doses suppress it [35,36]. Atomoxetine directly acts as an NMDAR inhibitor, but it also downregulates gene expression of the NMDAR subunits [37,38]. Another NMDAR inhibitor, D-serine, has shown promising results in reducing attentional lapses in rodents [39]. Considering the emerging role of genetic factors in methylphenidate treatment response [40], herein, we would like to focus on the molecular and genetic basis of NMDAR malfunction and the way it affects the ADHD course. NMDAR dysregulation might be an important mechanism underlying the development of the disorder, and may at least by part explain the heterogeneity of patient’s symptoms. A better understanding of the role of NMDAR in the disease pathogenesis may lead to the development of novel, targeted therapies that could bring relief to ADHD patients.

## 2. Characterization of the N-Methyl-D-Aspartate Receptors (NMDAR)

### 2.1. NMDAR Structure

**Figure 1:**
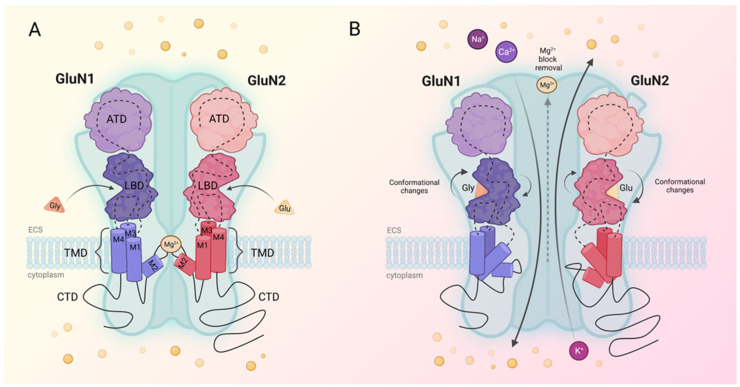
(**A**) The schematic structure of inactive GluN1/GluN2 N-methyl-D-aspartate receptor (NMDAR). Each subunit is composed of two extracellular domains: amino-terminal domain (ATD) and ligand binding domain (LBD); the transmembrane domain (TMD) consisting of three transmembrane helices (M1, M3, M4) and a re-entrant loop (M2); the intracellular carboxy-terminal domain (CTD). (**B**) The binding of glycine (Gly) to the GluN1 subunit and glutamate (Glu) to the GluN2 subunit triggers the closure of the ABD clam-shell around the agonists. This induces a conformational change in the linker and transmembrane helices, ultimately leading to the magnesium block removal. This in turn leads to an influx of sodium and calcium ions into the cytoplasm and an efflux of potassium ions into the extracellular space (ECS).

NMDARs are tetra-heteromers composed of the GluN1, GluN2, and GluN3 subunits. The subunits of NMDAR include two mandatory GluN1 subunits and, depending on the specific anatomical location within the brain, an additional two GluN2 or a combination of GluN2 and GluN3 subunits. There are four different types of GluN2 subunits (GluN2A, GluN2B, GluN2C, GluN2D) that can combine with the GluN1 subunit to form diverse receptor configurations [41]. Each of the mentioned subunits has a specific modular architecture with four semi-autonomous domains. The extracellular amino-terminal domain (ATD) is connected to the extracellular ligand binding domain (LBD). LBD is attached to the transmembrane domain (TMD), which forms the ion channel. TMD consists of three transmembrane helices (M1, M3, M4) and a re-entrant loop (M2), of which M1, M2 and M3 are coupled with LBD [42]. The last domain, the carboxy-terminal domain (CTD or C-tail), is localised intracellularly and is the most variable in terms of amino acid sequence [43]. Activation of the receptor, associated with removing the magnesium block from the channel and increase in calcium permeability, requires binding of a ligand and prior membrane depolarisation. Glutamate, the NMDAR agonist, binds to the GluN2 subunit, but additional binding of co-agonist glycine to the GluN1 subunit is required for efficient opening of the channel [44]. The binding of both glutamate and glycine closes the ABD clam-shell around the agonist, followed by a conformational change in the linker and transmembrane helices, and opening of a cation-selective pore [45]. As a prior membrane depolarisation is also essential for NMDAR activation, the receptor is usually localised closely to another ionotropic glutamate receptor, namely the α-amino-3-hydroxy-5-methyl-4-isoxazole propionic acid receptor (AMPAR). Glutamate released from the presynaptic membrane activates the AMPAR, which allows for sodium ion influx and fast excitatory postsynaptic currents. If the intensity of the influx is sufficient for the voltage-dependent magnesium block to be removed, NMDAR permits calcium influx, which constitutes one of the essential mechanisms involved in synaptic plasticity [46] (Figure 1). 

### 2.2. Long-Term Potentiation (LTP) and Long-Term Depression (LTD)

**Figure 2:**
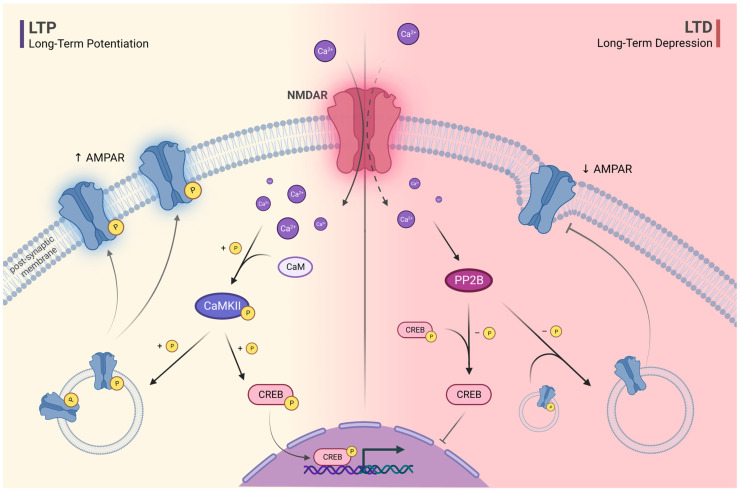
The schematic representation of N-methyl-D-aspartate receptor (NMDAR)-dependent long-term potentiation (LTD) and long-term depression (LTD). High calcium influx through NMDARs along with calmodulin (CaM) induce phosphorylation of calcium-calmodulin-dependent protein kinase II (CaMKII), which triggers LTP. CaMKII phosphorylates the subunit of α-amino-3-hydroxy-5-methyl-4-isoxazole propionic acid receptor (AMPAR), which leads to increased receptor concentration in the postsynaptic membrane. CaMKII also induces cAMP response element-binding protein (CREB) phosphorylation. Low calcium influx activates serine/threonine-protein phosphates 2B (PP2B). PP2B dephosphorylates CREB and AMPAR subunit, thereby facilitating LTD.

Two extensively studied NMDAR-dependent forms of synaptic plasticity associated with learning and memory are long-term potentiation (LTP) and long-term depression (LTD). As both LTP and LTD are induced by NMDAR-dependent calcium influx, the suggested differentiating factor is the level of activity or depolarisation in the postsynaptic cell. In contrast to LTP, which is triggered by significantly stronger NMDAR activation and a larger increase in postsynaptic calcium influx, LTD is triggered by modest NMDAR activation and modest increase in postsynaptic calcium influx [47]. Another hypothesised differentiating factor is the adequate synchronisation of presynaptic and postsynaptic neurons activity potentials. LTP can be induced if the presynaptic membrane is repeatedly depolarised shortly before the postsynaptic membrane, whereas LTD is triggered when a postsynaptic neuron is stimulated first [48]. In both NMDAR-dependent postsynaptic LTD and LTP, NMDAR heteromers containing GluN2A and GluN2B play a critical role [49]. In LTP, high calcium influx from the extracellular space activates multiple signalling molecules within postsynaptic cells, including calcium–calmodulin-dependent protein kinase II (CaMKII) [50]. CaMKII phosphorylates GluA1, the subunit of the AMPAR, which ultimately leads to an increased density of AMPAR in the postsynaptic membrane [51]. Activated CaMKII also induces cAMP response element-binding protein (CREB) phosphorylation. CREB is a transcription factor that regulates expression of numerous neuropeptides and is considered one of the most important factors involved in neuroplasticity and long-term memory formation. Conversely, in LTD, low calcium influx activates serine/threonine-protein phosphatase 2B (PP2B, also known as calcineurin), which dephosphorylates both CREB and the AMPAR GluA1 subunit [52,53]. Consequently, AMPAR are removed from the synapses and internalised during LTD. However, molecular mechanisms leading to LTD are more complex and not fully understood [54]. NMDAR activation during LTD can locally trigger caspase-3 activation via the mitochondrial pathway. Although global activation of caspases leads to apoptosis, restricted and localised activation may be essential for physiological, non-apoptotic mechanisms, such as synaptic remodelling and dendritic shrinkage [55]. In a mouse model, NMDAR stimulation led to rapid and temporary cytochrome c release from mitochondria [56]. Caspase 3-deficient neurons in culture did not exhibit spinal contraction in response to NMDAR stimulation. Moreover, caspase-3 knockout mice showed increased spinal density and synaptic strength, consistent with a critical role for caspase-3 in LTD. As there is a correlation between NMDAR malfunction and alterations in caspase-3 levels [57], it is not surprising that caspase-3 deficient mice exhibited inattention, impulsivity, and decreased ability to habituate to novel stimuli [58]. LTP and LTD are crucial mechanisms of synaptic plasticity involved in memory function in adults, but they are also involved in early brain development [59,60]. Interestingly, exposure to NMDAR antagonists during developmental stages in mice caused specific behavioural symptoms, resembling ADHD [61] (Figure 2).

LTP and LTD modulate vastly interconnected neuronal pathways providing a neurochemical foundation for learning and memory. Alterations in synaptic plasticity can lead to structural changes in neuronal networks, contributing to the cognitive and behavioural deficits observed in ADHD. Although the role of synaptic plasticity in ADHD is not fully elucidated, several genes implicated as risk factors for ADHD are linked to synaptic plasticity and neurodevelopment [62,63]. Herein, we present a concise overview of selected ADHD-associated genes and their involvement in synaptic plasticity (Table 1).

## 3. Genetic Factors Related to NMDAR Dysfunction and Developing ADHD

### 3.1. GRIN Gene Variants and Expression Profile

The *GRIN* gene family encodes the following classes of NMDA receptor (NMDAR) subunits: the glycine-binding GluN1, which is the product of *GRIN1*, glutamate-binding GluN2, which has 4 paralogs (A-D) encoded by *GRIN2A, GRIN2B, GRIN2C*, and *GRIN2D*, respectively, and the glycine-binding GluN3, encoded by *GRIN3A* and *GRIN3B* [45]. GluN2A and GluN2B subunits are highly expressed in the cerebral cortex and hippocampus, and to date they have been the most well-studied [72]. There are significant physiological differences between NMDAR containing GluN2A and GluN2B, such as different opening probability, which is higher for GluN2A-NMDAR [73]. Furthermore, since both glutamate and glycine are less potent at GluN2A compared to GluN2B, the deactivation time course following the removal of glutamate is shorter for GluN2A-NMDAR [74,75]. Physiologically, *GRIN2* gene expression changes throughout the developmental stages. The GluN2B subunit is strongly expressed during the prenatal phase, and then its expression drops during postnatal stages. When it comes to GluN2A, its expression appears to be low during the prenatal period and rises after delivery [76,77]. Presumably, the main mechanism responsible for the changes seen in protein and gene expression of NMDAR subunits is DNA hypermethylation [78]. One of the identified factors inducing dysregulation in the *GRIN2* gene expression is exposure to methamphetamine. In rodents, repeated administration of methamphetamine led to a decrease in *GRIN2A* expression in the hippocampus and decreased *GRIN2B* expression in the striatum [79]. Rats prenatally exposed to methamphetamine are considered a suitable animal model for ADHD, presenting symptoms like hyperactivity or memory malfunction [80,81]. Another factor influencing the *GRIN* gene expression is prenatal nicotine exposure (PNE) [82]. PNE is a well-studied ADHD risk factor, supported both by animal research [83,84] and by cohort studies [85,86]. All things considered, there may be a link between ADHD and altered *GRIN* gene expression, but further research is needed to confirm the association.

Numerous variants and mutations of the *GRIN* genes have been found in patients with diverse neuropsychiatric disorders, including ADHD [87,88]. However, ADHD is not the most frequent condition among patients harbouring *GRIN* mutations. Intellectual disability, epilepsy, and autism spectrum disorder are all much more common in *GRIN*-mutant patients, but certain *GRIN* mutations are still strongly linked to ADHD [89]. One example of this is *GRIN2A* gene variants that were the first NMDAR-related genes associated with an increased risk of ADHD [90,91]. Research on single nucleotide polymorphisms (SNPs) in 205 families revealed a connection between specific *GRIN2B* variants and ADHD [92]. Mutations were found in all domains (ATD, ABD, TM, and CTD), but most frequently in the ABD and TM regions [46]. Mice with experimentally introduced *GRIN2A*(N615S) mutation showed hyperactivity and dysregulated attentional levels. Since the asparagine amino acid residue GluN2A(N615) controls the magnesium block, it is suggested that symptoms caused by the *GRIN2A*(N615S) mutation may result from magnesium block suppression and enhanced calcium permeability [93]. In the case study of 5 children with de novo *GRIN2B* mutations, behavioural tests showed prominent hyperactivity, impulsivity, distractibility, and a short attention span. Patients with ADHD-resembling phenotype carried the following *GRIN2B* mutations: t(9;12)(p23;p13), t(10;12)(p21;p13), (p.R682C) in ABD, (p.A636P) in M3 domain and (p.T268SfsX15) in ATD [94,95]. Other identified polymorphisms in patients with increased inattention measured in the Continuous Performance Test (CPT) are rs2229193 in *GRIN2A* and rs2284411 in *GRIN2B* [96]. rs2284411 could be pharmacologically relevant, since children with that polymorphism showed significantly better treatment responses to methylphenidate [97].

### 3.2. SorCS2 Gene Variants

*SorCS2* (sortilin-related VPS10 domain-containing receptor 2; chromosome 4) is a large gene composed of 30 exons, belonging to the VSP10p (Vacuolar Protein Sorting 10 protein)-domain receptors gene family, which encodes SorCS2 protein. SNPs in this gene have been associated with a multitude of neuropsychiatric disorders including bipolar disorder [98], schizophrenia [99], and ADHD [100]. Interestingly, in a Genome-Wide Association Study (GWAS) conducted on adult ADHD patients, SNP in the 1 intron of *SorCS2* gene rs4689642 has been recognised as the most relevant ADHD-associated polymorphism [100]. Furthermore, the study conducted on monozygotic twins discordant for ADHD has shown that *SorCS2* gene methylation (thus silencing) leads to reduced grey-matter volume in precentral and posterior orbital gyri, which induces symptoms of ADHD in affected children [101]. Therefore, better understanding of the role of SorCS2 at the molecular level can provide a novel insight into not well-known ADHD aetiology.

VSP10p act as sorting receptors and regulators of neuronal viability and function by controlling the intracellular trafficking of targeted proteins [102]. Both constituents of VSP10p, Sortilin and SorCS2, can form a complex with neurotrophin receptor p75NTR. The complex is required to control the release and function of pro-neurotrophins, such as pro-BDNF (pro-Brain-Derived Neurotrophic Factor) and pro-NGF (pro-Nerve Growth Factor). The mentioned pro-neurotrophins are precursors for the respective neurotrophins, BDNF and NGF, essential for the promotion of neuronal survival, death and synaptic plasticity [102,103]. The deficiency of SorCS2 disrupts the formation of the SorCS2-p75NTR complex, leading to a decrease in the release of BDNF. Furthermore, the absence of SorCS2 impairs the neuron’s capacity to respond to BDNF through the binding of its receptor—TrkB. Correspondingly, *SorCS2*-deficient mice presented impaired LTP, LTD, neurite outgrowth, and dendritic spines formation. Lack of *SorCS2* abolished NMDAR-dependent synaptic plasticity in the mouse hippocampus, resulting in a phenotype similar to ADHD. *SorCS2*-deficient mice displayed impairment of long-term memory formation and higher tendency to take a risk and stimulus-seeking behaviour, however, this was accompanied by higher stress vulnerability [69]. Furthermore, studies have shown that SorCS2 acts as a selective regulator of NMDAR trafficking towards the surface of hippocampal neurons, as well as regulating dendritic spines density (synaptic plasticity) in pyramidal neurons of CA2 region. In the same study, *SorCS2*-deficient mice exhibited significant social memory deficits, however, without abnormalities in other hippocampal-dependent behaviours [104]. In addition, the SorCS2-p75NTR complex is also considered an essential regulator of development of dopaminergic projections. The lack of any complex subunits resulted in reduced dopamine levels and metabolism, as well as dopaminergic hyperinnervation of the frontal cortex. Interestingly, as the combined effects of dopaminergic dysregulation are associated with abnormal response to psychostimulants, administration of amphetamine on double knockout models displayed a paradoxical calming response [105]. Furthermore, *SorCS2*-deficient mice exhibited an altered dopaminergic firing pattern within the ventral tegmental area (VTA). The dopaminergic transmission was shifted from an irregular to a more regular pattern, along with an associated change in dopaminergic receptor sensitivity (namely, decrease in D1 and increase in D2 sensitivity). Behaviourally, mice presented a general reward deficit, novelty-induced hyperactivity, and yet paradoxical tranquillity in response to amphetamine—a phenotype reminiscent of ADHD [106].

### 3.3. D4.7R Variant

There are five subtypes of dopamine receptors: D1, D2, D3, D4, and D5. The D1 and D5 dopamine receptors belong to the D1-like family, whereas the D2, D3, and D4 receptors belong to the D2-like family [107]. The D4 receptor is responsible for signalling in the mesolimbic pathway, which takes part in several cognitive processes, such as motivation, desire for rewards, reinforcement learning, and emotional regulation. D4 is encoded by the *DRD4* gene located on chromosome 11 at 11p15.5 [108]. *DRD4* exhibits numerous polymorphisms in its coding sequence, and the most common polymorphism occurs in a region encoding the third intracellular loop of the receptor. This polymorphism results in variable number of tandem repeats of a 48-base pair sequence in the third exon [109,110]. The most common *DRD4* polymorphism products are D4.2, D4.4 and D4.7, characterized by 2, 4, and 7 repeats of a proline-rich sequence of 16 amino acids, respectively [109]. D4.7 has been associated with various psychiatric disorders such as ADHD, substance addiction, and personality traits associated with impulsivity [111]. In vitro studies have implied that the sensitivity of the D4.7 for dopamine is half that of the D4.2 and D4.4 [112], and this allele was found in 41% of ADHD patients and only 21% of the control group [111]. ADHD child patients with 7 repeated alleles exhibit more imprecise and impulsive responses on neuropsychological tasks [112]. Furthermore, mice that expressed the D4.7 variant showed enhanced exploratory and novelty-seeking behaviours, similar to the phenotypic trait of human ADHD. Mechanistically, D4R binds to the SH3 domain of postsynaptic scaffolding protein PSD-95, which is connected to the C-terminus of NMDA receptor subunits (GluN1) by the PDZ domain [113]. Activation of different variants of D4R, like D4.7, and D4.4 decrease the NMDAR function in the PFC at varying degrees. The activity of interconnected neurons in PFC, which are dependent on NMDAR and responsible for synchronised network activity, is more strongly inhibited by D4.7 compared to D4.4 [114]. Activation of D4.7 induces greater suppression of both GluN1/PSD-95 binding and NMDAR surface expression in neurons in comparison to D4.4 activation [113]. Presumably, inhibition of GluN1/PSD-95 binding causes NMDAR hypofunction, which leads to impairment of synchronous network activity and suppressed PFC activity, characteristic for ADHD patients [114,115]. Thus, D4.7R variant might be an attractive target in development of future therapies, as several studies have found a significant association between the various *DRD4* polymorphisms and better response to methylphenidate as compared to placebo [116,117]. However, studies on the impact of the D4.7 variant on the response to methylphenidate present conflicting findings. Some studies show no significant association [118], while others report a reduction in the response [119]. Factors such as sample size, population differences, and the presence of other genetic and environmental influences could have contributed to these discrepancies. Additionally, administration of D-cycloserine (partial NMDAR agonist) mitigated high novelty-seeking behaviour in D4.7-expressing mice, which emphasizes a link between NMDAR modulation and ADHD pharmacogenetics [113].

### 3.4. BAIAP2 Gene Variants

BAIAP2 (also known as IRSp53) is an abundantly expressed, postsynaptic adaptor protein. It is implicated in the regulation of actin filaments assembly during dendritic spines development, as well as the regulation of NMDAR-mediated synaptic transmission and LTP [120]. BAIAP2 is encoded by the *BAIAP2* gene, located on the chromosome 17. SNPs within the mentioned gene have been suggested to be involved in ADHD aetiology, as well as abnormal cerebral lateralisation, which is also associated with ADHD-related symptoms [121,122]. The hippocampal neurons of *BAIAP2*-KO mice showed a selective increase in NMDAR activity, however, without significant changes in AMPAR-mediated transmission. This was followed by a substantial increase in LTP and, functionally, deficits in learning, memory, and social interactions [120,123]. Interestingly, both direct and indirect inhibition of NMDAR normalised social interactions in *BAIAP2*-deficient mice [123]. Furthermore, even a moderate reduction in BAIAP2 level led to a significant increase in hippocampal NMDAR density [124]. Consistently, re-expression of BAIAP2 in *BAIAP2*-mutant mice resulted in the restoration of NMDAR-mediated synaptic transmission and proper NMDAR/AMPAR ratio in the medial PFC region (mPFC). However, despite improvement in social interactions, the hyperactivity- and anxiety-like behaviour induced by *BAIAP2*-knockout were not rescued in such conditions [125]. Additionally, the deletion of *BAIAP2* suppressed neuronal firing variability and dynamics within excitatory mPFC neurons, especially in those encoding social information. Administration of NMDAR antagonist (memantine) restored burst firing in mPFC neurons and rescued social deficits [126]. Altogether, the mentioned data emphasize the key role of BAIAP2 in regulating NMDAR-dependent signal transduction and, as a result, proper neuropsychological function.

### 3.5. SNAP-25 Gene Variants

Synaptosomal-associated protein, 25 kDa (SNAP-25) is a part of the SNARE complex, which is involved in the exocytotic release of neurotransmitters during synaptic transmission. Furthermore, SNAP-25 plays an important role in modifying NMDAR and kainate receptor density in the postsynaptic membrane [127]. Protein kinase C (PKC)-mediated phosphorylation of SNAP-25 facilitates the transport of postsynaptic vesicles and their subsequent fusion with the plasma membrane, resulting in the insertion of NMDA channels onto the cell surface [128]. Since SNAP-25 affects NMDAR density in the postsynaptic membrane, it is not surprising that downregulation of SNAP-25 impairs LTP [129], which as a result affects synaptic plasticity and memory function. Although the actual mechanism by which SNAP-25 affects psychiatric disorders are not well known, numerous studies have shown a connection between alterations in SNAP-25 levels and symptoms of multiple mental disorders, including ADHD [130]. Symptoms such as spontaneous hyperactivity are seen in animals with *SNAP-25* deletion, known as coloboma mice [131,132]. Mice with a knockout of one of the *SNAP-25* genes (complete knockout is lethal) present mild hyperactivity [133]. The hyperactive phenotype observed in mentioned animals has driven the search for *SNAP-25* mutations associated with ADHD among humans. Barr et al. genotyped DNA from 122 patients diagnosed with ADHD and found two significant mutations, the MnlI polymorphism and DdeI polymorphism [134]. Both haplotypes showed biased paternal transmission to affected probands [135,136]. More recently, some studies have suggested an association between microsatellite repeats within the *SNAP-25* and ADHD prevalence [137,138]. In one meta-analysis, four *SNAP-25* gene variants were confirmed as ADHD risk genes. These included: rs362987 on intron 4, rs363006 on intron 6, and aforementioned MnlI (3′UTR rs3746544) and DdeI (3′UTR rs1051312) [139]. A subsequent study found another polymorphism associated with ADHD, the rs362549 [140]. SNAP-25 polymorphisms influence ADHD severity [141,142]. The impact of the MnlI on symptom intensity in ADHD is the most well-known. Children with the MnlI gene showed significantly decreased local functional connectivity density (lFCD) in the ACC, as well as decreased lFCD in the dorsal lateral PFC [143]. Another study has found a correlation between altered working memory and carrying the MnlI gene [144].

*SNAP-25* gene variations might be an important predictor for methylphenidate response. The strongest association with pharmacotherapy was seen in children with MnlI polymorphism [145,146]. The mechanisms underlying different responses to treatment among patients with MnlI variants remain unclear, However, some studies suggest that changes in brain metabolite levels and in haemodynamics might play a key role in this phenomenon [147,148,149].

### 3.6. Latrophilin-3 Gene Variants

Latrophilin-3 protein (LPHN3p) is a brain-specific member of a small subfamily of adhesion G protein-coupled receptors. It is encoded by the *ADGRL3* gene (also known as *LPHN3* gene), located on chromosome 4. Functional studies have demonstrated that *LPHN3* variants were expressed mainly within brain regions associated with attention and activity (such as PFC, caudate, hippocampus, amygdala, and cerebellum) and were implicated in both ADHD development and pharmacogenetics [150,151]. Indeed, a multitude of studies has confirmed a crucial role of *LPHN3* SNPs in susceptibility to ADHD, as well as its predictive role in ADHD severity, associated comorbidities, and drug responsiveness [152,153,154,155]. However, the direct molecular mechanism underlying LPHN3p contribution to ADHD development have not yet been fully elucidated. LPHN3p, when combined with its endogenous ligands, acts as a crucial regulator of proper excitatory pyramidal neurons functioning, both in the neocortex and hippocampus. Mechanistically, LPHN3p regulates cortical and hippocampal glutamatergic synaptic formation and density [156,157,158]. Deficiency of one endogenous ligand of LPHN3p, namely Leucine-rich repeats transmembrane protein (FLRT3), resulted in significantly reduced NMDAR-mediated excitatory postsynaptic currents (EPSCs) within the hippocampus [131]. Furthermore, LPHN3-knockout mice demonstrated impairment of early LTP in the CA1 region of the hippocampus with concomitant reduction in NMDAR-GluN1 expression. This resulted in hyperactivity and hippocampal-mediated learning and memory deficits, characteristic of ADHD phenotype [159,160].

### 3.7. PCDH7 Gene Variants

*PCDH7* is a gene that belongs to the protocadherin gene family and encodes an extracellular protein Protocadherin 7 (PCDH7p). PCDH7p is an integral protein of plasma membrane, which plays role in cell–cell recognition and adhesion. Few variants in *PCDH7* have been identified as rare, but at the same time significant risk loci for ADHD development [161,162]. Interestingly, PCDH7p has been found to interact with the N-terminal domain of the GluN1 subunit of NMDAR. Consequently, PCDH7p overexpression resulted in a reduction in synaptic NMDAR current and impairment of dendritic spines morphology within the hippocampus, observed as collapse of spines and abnormal dendritic swelling [137]. On the other hand, knockout of *PCDH7* resulted in elongation of dendritic protrusions beyond typical spine size [163]. Altogether, *PCDH7* constitutes another potential factor affecting synaptic NMDAR function and ADHD risk; however, further research is required to validate this hypothesis.

### 3.8. Other Genes

Recent advances in high-throughput technologies have enabled mapping and holistic analysis of numerous genetic variants, leading to a more comprehensive understanding of molecular changes in normal development and disease. The utility of integrative approaches is particularly important for diseases such as ADHD, where genetic and environmental factors interact with each other [164]. Multi-omics analysis examines interconnections across genomics, epigenomics, transcriptomics, and metabolomics, aiming to elucidate the biological mechanisms behind ADHD and identify potential biomarkers. This approach allows us to determine how environmental factors (e.g., parental smoking, glucocorticoid exposure) influence the child’s genome, ultimately contributing to the manifestation of genetically correlated traits of ADHD phenotype (e.g., childhood aggression, insomnia, tendency to addiction) [165,166]. Furthermore, this approach can unveil new candidate genes implicated in the pathogenesis of ADHD. A multi-omics study by Cabana-Dominguez et al. has identified seven modules of co-expressed genes associated with ADHD. These modules consist of genes that are pivotal for the genetic and epigenetic control of neurodevelopment and immune response [167]. Among them a number of genes (e.g., SP3, CUX1) are involved in neuronal differentiation, synaptogenesis and synaptic plasticity. IQSEC1 is essential for the maintenance of glutamatergic synapses, while CNTNAP2 for neurocognitive development and neuron-glia interactions [167,168]. While certain research suggests the involvement of these genes in regulating NMDAR expression and glutamatergic signalling [169,170,171,172,173], there is a scarcity of studies focused on investigating these interactions comprehensively. Nevertheless, combination of multi-omics and mechanistic approaches might constitute a promising direction for future research.

## 4. Treatment Strategies Targeting NMDAR

Given the side effects of commonly used stimulant drugs in treating ADHD, there is a need to explore alternative therapies, especially for children. Hence, the use of drugs targeting NMDAR as a treatment for ADHD is an area of ongoing research.

Amantadine and memantine are both adamantane derivatives that act as non-competitive NMDAR antagonists. These drugs have been shown to stabilise NMDAR and inhibit prolonged calcium influx, which protects neurons from excitotoxicity while preserving normal synaptic activity [174,175]. Amantadine not only acts as an NMDAR antagonist but also affects dopaminergic neurons by increasing dopamine release and preventing its reuptake. On the other hand, memantine exhibits additional antagonist activity on the serotonin type 3 (5-HT3) and nicotinic acetylcholine receptors [176,177]. Both amantadine and memantine have shown promising results in improving ADHD symptoms with fewer side effects than methylphenidate in small, randomised, double-blind clinical trials. They are well-tolerated up to doses of 150 mg/day and 20 mg/day, respectively, with temporary appetite decrease being the most common side effect [178,179]. Memantine has been examined in adult patients both as monotherapy and as an adjustment to methylphenidate treatment [180,181]. However, these studies were conducted on small sample sizes and further randomised trials with larger numbers of participants are necessary to confirm their effectiveness. It is also worth noting that there are currently no medications for ADHD that specifically target NMDAR. As a result, pharmacotherapy for ADHD largely relies on stimulant or non-stimulant medications that target the dopaminergic and noradrenergic systems. However, as mentioned before, even commonly used stimulant medications like methylphenidate can affect NMDAR function [36].

There are also other FDA-approved drugs for the monotherapy treatment of ADHD, namely atomoxetine and guanfacine, which have been found to interact with NMDAR. Atomoxetine is a presynaptic norepinephrine reuptake inhibitor that also suppresses dopamine reuptake in particular brain regions, such as the PFC [182]. In the murine ADHD model, atomoxetine affects mRNA and protein levels crucial for synaptic plasticity in the hippocampus, with a particular interest in reducing the GluN2B subunit of NMDAR [38]. Additionally, studies have shown that atomoxetine re-established LTP in the hippocampus, further emphasizing its modulatory influence on synaptic plasticity [183]. Previous research indicated that guanfacine, an α-2 adrenoceptors (α-2AR) agonist, could prevent spatial working memory deficits caused by the NMDAR antagonist phencyclidine, suggesting that α-2AR could play a significant role in cognitive deficits related to NMDA receptor hypofunction [184]. However, a recent study found that guanfacine did not directly affect tonic NMDA currents in PFC [185]. Nonetheless, it may have an indirect influence on the NMDAR function, which warrants further investigation. The mechanisms by which drugs might exert their therapeutic effect upon interactions with NMDARs are summarized in Table 2.

It should also be kept in mind that various genetic mutations may differently affect the expression of NMDAR subunits and, in turn, exert different impact on response to treatment in ADHD patients, as presented in Table 3.

## 5. Conclusions

ADHD is a neurodevelopmental disorder commonly attributed to a complex interplay between genetic and environmental factors. Mutations and SNPs in numerous genes, including those associated with NMDAR, have been identified as genetic susceptibility factors for ADHD. These genetic variants have been shown to have regulatory effects on glutamatergic neurotransmission by regulating NMDAR function and distribution, NMDAR-dependent LTP and LTD, and synaptic plasticity. The most compelling evidence is provided by *GRIN* gene variants. Changes in the expression of *GRIN* genes directly impair the synthesis of NMDAR subunits and are strongly associated with an elevated risk of developing ADHD. In addition to *GRIN*, deficiencies in other genes associated with ADHD susceptibility, such as *SorC2*, *D4.7R*, *SNAP-25*, and *LPHN3*, have also been shown to result in NMDAR hypofunction. For example, *SorC2* deficiency leads to impaired NMDAR-dependent LTP, LTD, and synaptic plasticity. The *D4.7R* variant inhibits the GluN1 subunit of NMDAR via interaction with PSD-95. Downregulation of *SNAP-25* reduces NMDAR synaptic density and impairs LTP, whereas *LPHN3* knockout leads to early LTP impairment and reduced NMDAR-GluN1 expression. Conversely, *BAIAP2* deficiency has been found to increase NMDAR density and NMDAR-mediated synaptic transmission. Interestingly, the most used drugs for treating ADHD, such as methylphenidate and atomoxetine, have been shown to regulate NMDAR function. On the other hand, drugs directly targeting NMDAR, such as amantadine and memantine, have demonstrated effectiveness in alleviating ADHD symptoms in clinical trials.

It is noteworthy that, according to proton magnetic resonance spectroscopy studies, glutamatergic imbalance in the context of ADHD differs in terms of particular neural circuits and age. For example, studies in adult ADHD patients have demonstrated reduced levels of a glutamate marker in the medial PFC, ACC, and basal ganglia, while elevated in the cerebellum. On the other hand, studies with children and adolescents suggest increased glutamate markers in the frontal cortex and ACC [188]. Also, children with ADHD exhibit reduced GABA levels in the somatosensory/motor cortex and the striatum [14,189]. Both GABA and glutamate levels in the frontostriatal circuitry are age-dependent; this suggests different developmental trajectories of neurotransmitter imbalance, likely due to the complex interplay between genetic and environmental factors [18]. Genetic studies also highlight the involvement of excitatory and inhibitory neurotransmitter systems in the development of ADHD, with an emphasis on glutamate neurotransmission [190]. Furthermore, the crosstalk between glutamatergic and GABAergic synapses may contribute to the modulation of neuronal excitability and synaptic plasticity (reviewed in [191]), which underlines the role of pathological excitation-inhibition imbalance in ADHD. In addition, interactions between glutamatergic and dopaminergic signalling are worth considering as the prevailing theory of ADHD pathogenesis suggests that hyperactive and inattentive behaviours may arise from deficiencies in either tonic or phasic dopamine levels [192]. D1 and D2 receptors modulate striatal glutamatergic signalling and, vice versa, NMDAR is crucial for the regulation of midbrain dopaminergic neurons activity [193,194]. Altogether, these studies emphasize the need for a holistic interpretation of complex brain biochemistry in the pathogenesis of ADHD.

Although animal models offer valuable insights into mechanistic data on disease pathogenesis, they face limitations in direct translation of these findings to humans. Currently, there is a lack of substantial clinical data that directly establishes a connection between NMDAR dysfunction and ADHD in human populations. This draws attention to the exploration of non-invasive methods to bridge the gap between basic research and clinical applications. The implementation of advanced neuroimaging techniques and multi-omics studies can potentially aid in the identification of ADHD-related biomarkers (inter alia neurochemical signals, electroencephalography patterns, genetic variants, epigenetic markers, metabolomic profiles) [166,195]. These biomarkers could assist in diagnosis, treatment monitoring, and targeted interventions. Furthermore, advances in pharmacogenetic research could strengthen the genetic link between ADHD and NMDAR dysfunction and determine the therapeutic implications of specific genetic variants [Table 3]. Blood-based biomarkers, such as cytokines or microRNAs, could reflect systemic changes resulting from NMDAR dysfunction in the brain [196,197]. Future identification and validation of reliable biomarkers for NMDAR dysfunction in ADHD would not only support the diagnostic process but also drive the development of precision medicine approaches. However, designing a specific biomarker for NMDAR malfunction is a challenging task, as the receptors are highly heterogenous.

The NMDAR complex is highly polymorphic, with multiple subunits and splice variants that contribute to the functional diversity of the receptor. Genetic variations in NMDAR genes can influence receptor activity, subunit composition, and synaptic localisation, which in turn affects synaptic plasticity and neurotransmission. The expression of NMDAR subunits varies across different regions of the brain. For example, GluN2A and GluN2B subunits are predominantly expressed in the cortical regions and hippocampus, which are key brain regions involved in the ADHD development. Conversely, GluN2C and GluN2D expression is much weaker in the aforementioned regions [46]. It is thus presumed that preferential targeting of GluN1-GluN2A/B NMDARs would be a more appropriate approach in regard to alleviating ADHD symptoms, and these types of the receptor should be given the most attention in further studies. This, however, poses an issue of modulating NMDAR activity in a highly selective manner, which adds another layer of complexity to developing personalised treatment strategies for individuals with ADHD. It is not excluded that specific NMDAR modulators could be used in combination with currently available therapeutic strategies, but first, such drugs need to be selected by high-throughput screening or related approaches and tested in preclinical models. As studies on NMDAR in animal models presented herein mostly relied on genetic interventions, these findings should also be validated by pharmacological approaches before clinical implementation. On the other hand, FDA-approved drugs that modulate NMDAR activity should be extensively tested in double-blind, randomised trials in larger cohorts to fully assess their potential effectiveness. Moreover, the potential inhibition of NMDAR activity raises questions about the effects on other brain pathways and intracellular signalling, which may lead to potential adverse effects. It is important to emphasise that therapeutic approaches must moderately regulate the activation of NMDAR activation so as not to induce excitotoxicity, especially in the context of altered neurotransmission dynamics in ADHD pathogenesis. Excitotoxicity is characterised by an excessive influx of calcium ions, leading to the production of reactive oxygen species and the activation of apoptotic pathways, and resulting in neuronal damage and cell death [198]. Considering the potential vulnerability of individuals with ADHD to excitotoxicity [199], therapeutic strategies targeting NMDARs should aim to restore normal receptor activity without inducing excitotoxic effects.

Taking all of this into consideration, attempts to treat ADHD by targeting the NMDA receptor may pose significant challenges. However, gaining a better understanding of its role in the disease development could shed a new light on the complex pathogenesis of ADHD and lead to development of novel therapeutics.

## Figures and Tables

**Table 1:** Genes modulating synaptic plasticity and their impact on attention deficit hyperactivity disorder (ADHD) symptoms.

Genes	Role in Synaptic Plasticity	Implications for ADHD
BDNF	BDNF regulates excitatory and inhibitory synapse formation, LTP and LTD.	Variations in *BDNF* are associated with increased hyperactive-impulsive symptoms and learning deficits in ADHD [64]
SLC6A2	SLC6A2 is involved in norepinephrine reuptake, regulating noradrenergic system at the synaptic cleft.	*SLC6A2* variations can alter norepinephrine levels, affecting synaptic potentiation and contributing to ADHD symptoms [65]
SLC6A3	SLC6A3 is involved in dopamine reuptake, affecting synaptic neurotransmitter levels.	Variations in *SLC6A3* can lead to altered dopamine levels, impacting synaptic potentiation. ADHD-associated genetic variants in *SLC6A3* are linked to disrupted cortical thickness and potential synaptic dysfunction [66,67]
GRM family, SORCS2	Members of GRM family and SORCS2 regulate LTP and LTD.	Dysregulation of glutamate signalling due to variations in *GRM genes* and *SORCS2* contributes to abnormal synaptic connections in ADHD, potentially leading to cognitive and behavioural symptoms [68,69]
5-HT1B, SLC6A4	Serotonin receptor 5-HT1B and serotonin transporter SLC6A4 play roles in synaptic potentiation.	Dysregulation of serotonin signalling, as seen in ADHD-associated genes, can affect glutamate release and synaptic potentiation, contributing to ADHD symptoms [70]
NOS1	Affect the strength of synaptic transmission and contribute to plasticity mechanisms such as LTP and LTD.	Dysfunctional *NOS1* signalling may contribute to ADHD-related behavioural traits such as impulsivity and deficits in spatial learning [71]

Abbreviations: ADHD—attention deficit hyperactivity disorder; LTP—long-term potentiation; LTD—long-term depression; *BDNF*—brain-derived neurotrophic factor; *SLC6A2*—sodium-dependent noradrenaline transporter; *SLC6A2*—sodium-dependent dopamine transporter; *GMR family*—glutamate metabotropic receptor family; *SORCS2*—suppressor of cytokine signaling 2; *5-HT1B*—5-hydroxytryptamine receptor 1B; *SLC6A4*—sodium-dependent serotonin transporter; *NOS1*—nitric oxide synthase 1.

**Table 2:** Pharmacological interventions targeting n-methyl-d-aspartate receptor (NMDAR) for attention deficit hyperactivity disorder (ADHD) treatment.

Drug	Target	Functional Changes
Amantadine	NMDAR antagonist ↑ Dopamine release ↓ Dopamine reuptake	↑ Rate of channel closure ↓ Prolonged calcium influx Protects against excitotoxic neural injury [174]
Memantine	NMDAR antagonist ⊣ 5-HT3-R ⊣ nAChR	⊣ Extrasynaptic NMDAR-mediated currents Preserves normal synaptic activity Protects against excitotoxic neural injury [175]
Atomoxetine	⊣ NET ↓Dopamine reuptake	↓ mRNA and protein levels of NMDAR’s GluN2B subunit [38] Modulates synaptic plasticity in the hippocampus [183]
Guanfacine	α-2AR agonist	Prevents NMDAR antagonist-induced spatial memory deficits [184]

Abbreviations: NMDAR—N-methyl-D-aspartate receptor; ↑—increases; ↓—decreases; ⊣—inhibits, 5-HT3-R—serotonin type 3 receptor; nAChR—nicotinic acetylcholine receptor; NET—presynaptic norepinephrine transporter, α-2AR—α-2 adrenoceptor.

**Table 3:** The effect of selected polymorphisms in ADHD-susceptibility genes on response to conventional pharmacological treatment.

Gene	ADHD-Associated Variants	Effect on NMDAR Function	Treatment Results	Model
GRIN2B	rs2284411	Impaired GluN2B expression	↑ MPH response	Children and adolescents with ADHD [97]
DRD4	D4.7R	Reduced GluN1 expression	↑/↓ MPH response	Children with ADHD [116,117]
			↑ D-cSer response	D4.7-expressing mice [113]
SNAP-25	MnIl		↑ MPH response	Children and adolescents with ADHD [146]
		Impaired NMDAR trafficking		
	Ddel		↑/↓ MPH response	Pre-schoolers with ADHD [145]
LPHN3	rs6551665	Reduced GluN1 expression	↑/↓ MPH response [127,150]	Children with ADHD [150,186]
	rs6858066 rs1868790		↑ MPH response	Children with ADHD [186] Children and adolescents with ADHD [187]

Abbreviations: MPH—methylphenidate; D-cSer—D-cycloserine; ↑– better treatment results in comparison with a group without certain polymorphism; ↓—worse treatment results; ↑/↓—conflicting or unobvious findings on response to treatment.

## Data Availability

Not applicable.
